# Yellow meconium

**DOI:** 10.1007/s12024-024-00932-2

**Published:** 2024-12-21

**Authors:** Johanna Preuß-Wössner, Jan-Peter Sperhake, Burkhard Madea

**Affiliations:** 1 University Hospital Schleswig-Holstein, Institute of Legal Medicine, Kahlhorststr. 31-35, Building 89, Campus Kiel: Arnold-Heller-Str. 3, Building 28, 23562, 24105 Lübeck, Kiel, Germany; 2 Institute of Legal Medicine, University Medical Center Hamburg-Eppendorf, Butenfeld 34, 22529 Hamburg, Germany; 3 Bonn, Germany

**Keywords:** Meconium, Color, Vitality, Putrefaction

## Abstract

The intestinal content of newborns is known as meconium, usually described as a black-greenish slimy substance. However, in rare cases, meconium might be yellow. This is of great relevance with regard to the forensic assessment of life birth, i.e. (longer) survival time and possible (breast-)feeding before death. While in older German forensic literature, yellow meconium has been occasionally mentioned from the 19th century on - without any conclusions being drawn from this finding whether the newborn was alive or had been fed -, current forensic literature does not provide information about this phenomenon. No reports of yellow meconium were found in (older) English forensic literature as well. We report two cases of newborn autopsies with yellow meconium and provide a short overview of the respective literature.

**Supplementary information:**

The online version contains supplementary material available at 10.1007/s12024-024-00932-2.

## Case report

In 2020, the putrefied body of a male newborn was found on a private property stored outside in a sealed bag. The newborn’s mother was identified and subsequently accused of involuntary manslaughter. The mother had hidden her pregnancy and plausibly claimed a birth 10–11 months before the body was found, although the storage conditions remained unclear.

**Fig. 1:**
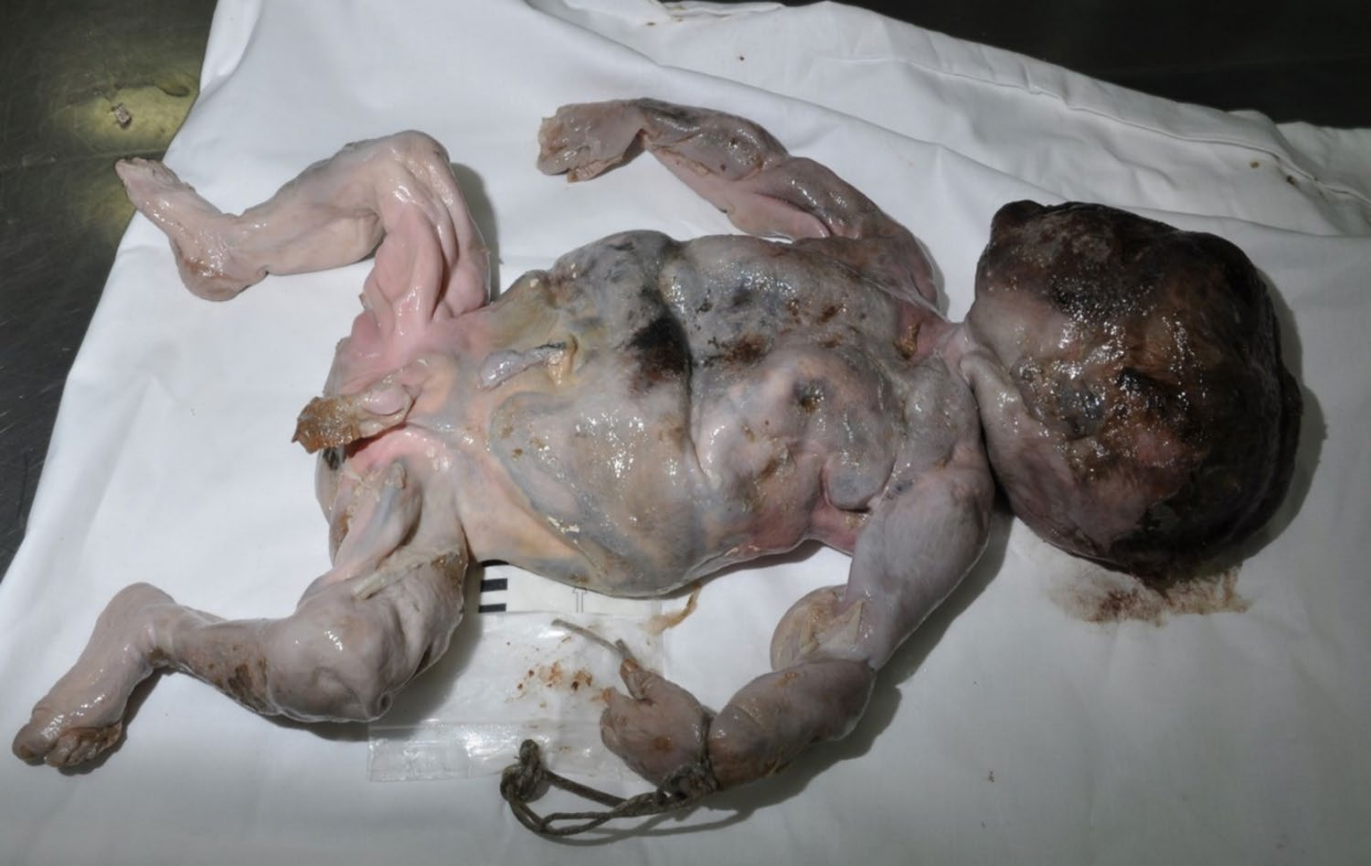
Newborn corpse of the first case

**Fig. 2:**
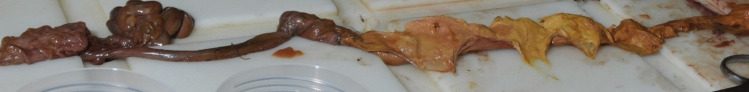
Colon contents, case 1

Due to putrefaction it was not possible to determine at autopsy whether the child had lived after birth or not (Fig. 1). Strikingly the entire large intestine was filled with voluminous golden-yellow content and did not contain any ‘typical’, i.e. black-greenish meconium (Fig. 2). This finding was interpreted as an indication that the child had been fed with (breast) milk after birth and meconium had already left the body, meaning that the child had been alive for a certain period of time.

By request of the lawyer of the mother, an intensive literature research was performed with respect to possible other explanations of the golden-yellow color of the meconium, especially in the older literature, which revealed astonishing results (Table 1).

**Table 1:** Sources from the German-language specialist literature

Author	Text (translation by the authors)
Krahmer 1857 [8]	… in my examinations of newborn children I have found the child's vomit in the transverse colon to be yellowish white, coloured like a mixture of milk and bile, rather than the usual bright leek green. ... The children were stillborn and had not ingested any food through their mouths
Hecker and Buhl 1861 [4]	The child's saliva once had a strikingly light yellow color and consisted of fat, epidermal cells, woolly hairs, very little bile pigment and mucus.
Huber 1884 [7]	…with regard to the color of the meconium [] it should be noted that it is everywhere described as black-green. However, Buhl already mentions ... a meconium which was light yellow, contained fat, epidermis and woolly hairs and showed very little bile pigment and mucus.
Strassmann F 1895 [15]	It happens that the bile pigment content of the meconium is lower, which is why it shows a more light yellow color, especially in the upper parts of the large intestine (Krahmer), which is not due to the possible introduction of milk.
Schmidt 1897 [14]	… the black-green the end product of the yellow-brown, after it has been thickened by partial absorption of the water it contains and the cells have mostly shrunk and partly shattered
v. Hofmann 1903 [5]	…that not infrequently the meconium only shows the familiar dark green color in the lower sections of the large intestine and a yellowish-brown color in the upper sections, especially in the ascending branch…
Walz 1906 [17]	Huber distinguishes between two types of meconium, the Meconium amnioticum, of yellow-brown color, which is found in the upper intestinal sections and preferably contains the components of the swallowed amniotic fluid, and the Meconium hepaticum, which contains abundant mucus, bile pigment and intestinal epithelia. However, our case shows that even without an admixture of amniotic fluid components, the meconium in the upper intestinal sections can be yellowish and the dark green color apparently only occurs in the lower intestinal sections.
Haberda 1911 [2]	During the internal examination, as is well known, the evidence of the food eaten is also important for the diagnosis of how long the child has lived. In this respect, a deception could possibly be made, as the child's vomit in the upper parts of the large intestine is yellow in color, so that it looks like milk stool. It is well known that the puerperal feces in the upper colon is almost always lighter in color than in the lower colon, but the color is clearly green.
Haberda 1923 [3]	If the contents of the stomach or intestines show that the child has already been fed, then of course the assumption that the child died immediately after birth must be dropped, but one is not entitled to conclude from this finding alone that the child lived for several days, since it could have been fed in the first hour after birth. ... Exceptionally, the child's vomit in the upper third or even in the upper half of the large intestine may be brownish-yellow in color and simulate milk stool. In one case of infanticide by strangulation known to us, the yellow color of the meconium led to the false assumption that the child was not a newborn. Otherwise the color is dark green, in the lower part of the large intestine black-green
Strassmann G 1931 [16]	In several of the exhumed cases, the yellowish discoloration of the child's saliva, which Haberda had already pointed out earlier, was striking. However, as one sees again and again when examining post-mortem reports, the inexperienced post-mortem examiner still regards this as proof that the child had ingested food, i.e. milk. This yellowish discoloration of the child's sputum seems to me to be a not uncommon occurrence in the case of severe putrefaction of the corpse and can also occur in the case of artificially produced putrefaction of newborn corpses. Microscopically, normal components of puerperal excrement can be found in such yellow puerperal excrement, but the meconium bodies are often strikingly pale, colorless, and the dye that has leaked out of them can be seen in the surrounding area. These are obviously pure putrefaction processes and the conclusion from the yellow color of the child's feces in the large intestine that milk formula has been ingested is incorrect if no microscopic examination of the intestinal contents has taken place and provided evidence of the food ingested
Holzer 1940 [6]	Meconium, or puerperal excrement, the contents of the large intestine of the human fetus and the newborn, from the fifth month of pregnancy onwards with the characteristic green color caused by bile, can occasionally be brown-yellow in the upper part of the large intestine and simulate a milky stool (Haberda). G. Strassmann repeatedly found a conspicuous yellow coloration of the meconium, apparently caused by putrefaction processes, in exhumed corpses of newborns and warns against confusion with milk stools.
Ponsold 1950 and 1967 [9, 10]	Special care should be taken with rotting corpses. The black-green color of the child's saliva can quickly change to a golden-yellow color under the influence of putrefaction. This can lead to the misconception that the newborn was initially nourished and only later killed.
Reimann et al. 1985 [13]	Puerperal excrement: (meconium) black, paste-like, can become lighter in color post-mortem due to putrefaction
Forster 1986 [1]	The color of the meconium (also known as “child saliva”) is greenish-blackish. However, one should not be deceived by lighter colors and assume that food has been ingested: It may be due to bacterial action!

**Fig. 3:**
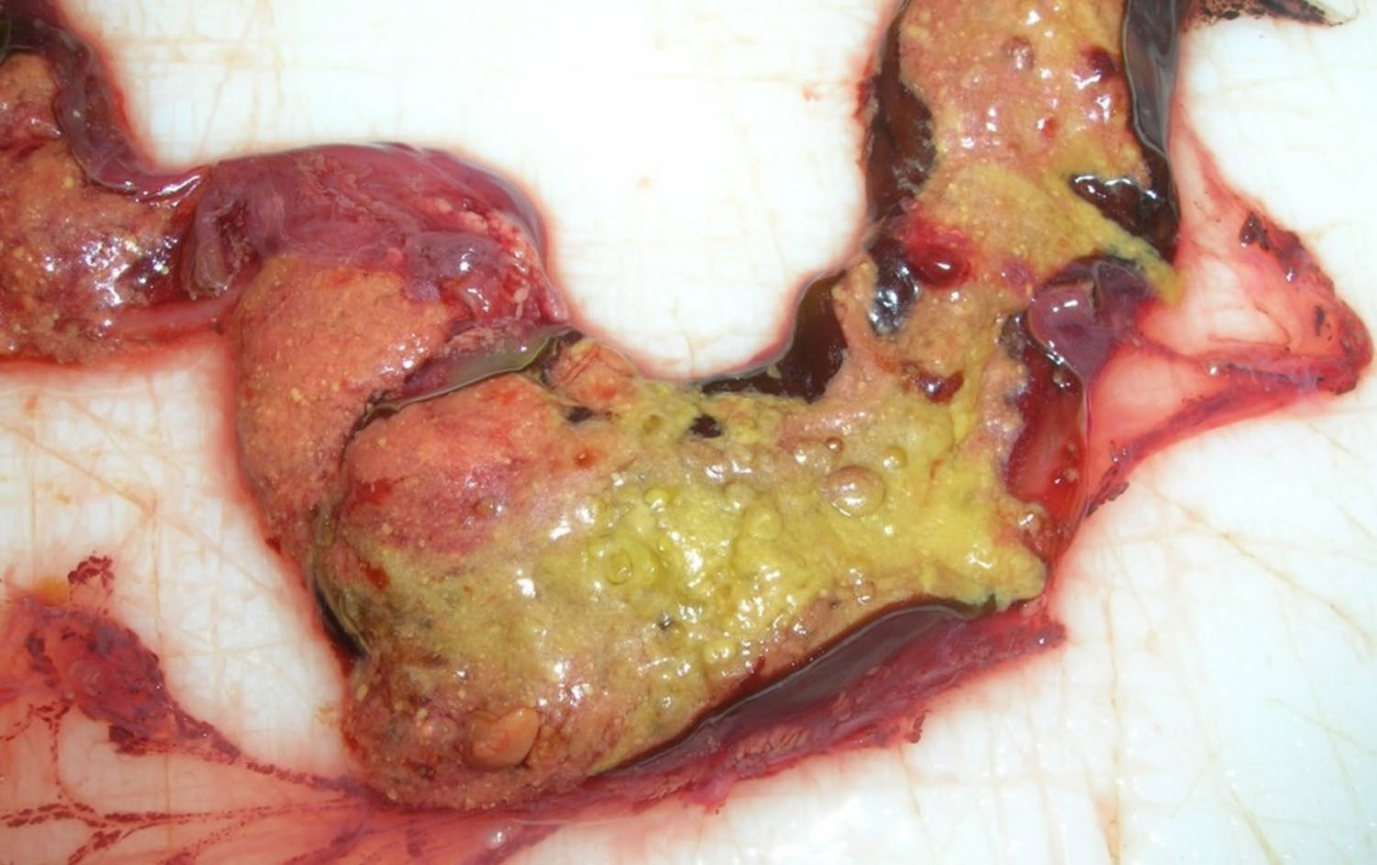
Colon contents, case 2

While this research was still ongoing, a stillborn child was subject to autopsy in the same Institute of Legal Medicine. The mature child had normal cardiotocogram at the onset of labor shortly before birth, but had then been delivered lifeless and could not be resuscitated. At autopsy two days post-mortem, the body of the newborn showed the onset of putrefaction of the upper abdominal organs and abundant yellow meconium in the entire large intestine (Fig. 3).

## Discussion

The main finding from these case reports and the literature research is that meconium is not always black-greenish. The newborn’s colon contents can also be golden yellow. This might happen without feeding and / or longer survival time and look like typical breast milk feces. It is rare, but it does happen [1, 2]!.

Intensive literature research, particularly in the older German forensic literature, showed that the knowledge of the possibility of the yellow color of the meconium was quite widespread in the 19th and 20th century. Nonetheless, this finding was never used to draw any conclusion about the child’s possible survival time or food intake / nutrition. The various sources in the German forensic literature of the last nearly 200 years are summarized in Table 1 [3–17]. No comparable description of yellow meconium could be found in the English forensic literature of the 20th and 21st century; reviewed English references are provided as Electronic Supplemental Material (ESM). In many English books the term “meconium” does not even appear in the index, or at most “meconium aspiration”, “meconium staining”, or “meconium aspiration syndrome”.

The rare occurrence of yellow meconium plausibly explains why this finding has fallen into oblivion - it was simply no longer reported due to its low frequency itself and declining number of neonatal autopsies. Compared to today, forensic pathologists in the second half of the 19th and first half of the 20th century encountered a relatively high number of neonatal autopsies. At the end of the 19th resp. the beginning of the 20th century, newborns made up 20% of all forensic autopsies [15].

A reason for the deviating color up to the very impressive golden yellow color of the meconium could not be identified with certainty. In 1931, Georg Strassmann was the first to assume a connection between yellow meconium and putrefaction [16]. He reported a total of 6 cases of exhumed newborns, all of which he examined within *one* year (1929). In two of these cases he described conspicuously yellowish infant saliva, in one case yellow-green saliva. The corpses were buried without a coffin between 16 days and 4.5 months, leading to a wide range of putrefaction and weakening the correlation between yellow meconium and decay. G. Strassmann also described histological findings of the yellow meconium: It contained normal components of the child’s saliva, but the meconium bodies were conspicuously pale, colorless and with the dye leaking from them in the surrounding area. From this, he concluded the connection with putrefaction. This was not questioned until 1986 - the last source before the current ‘rediscovery’ of this finding - without any further concrete reference or other evidence in favor of this hypothesis [3].

 Many of the historical references cited describe that the yellow meconium was mainly observed in the upper parts of the large intestine. This differentiation is only lost in the literature with G. Strassmann, after whom he considered the connection with putrefaction to be possible. Analogous to the historical references, it may also be considered a rule that the fecal matter is often only dark green in the lower sections of the large intestine and yellowish-brown in the upper Sect. [14].

The black-greenish color of the meconium is due to the bile pigments, especially biliverdin as an intermediate product of haem degradation. However, it seems obvious and understandable that the deviating (light) yellow or golden yellow color of the meconium is caused by a reduced proportion of bile pigments, maybe associated with a (temporary? ) reduction in the patency of the newborn’s bile ducts - for whatever reason. A connection with the content of bile pigments also seems plausible. Unfortunately, in the cases described here, the bile ducts were not explicitly described (in the first case due to putrefaction).

Social circumstances and developments can have a considerable influence on forensic autopsy routine. The declining number of autopsies of newborns is undoubtly absolute positive but must not lead to the loss of previously widely available empirical knowledge - and to incorrect assessments.

## Electronic Supplementary Material

Below is the link to the electronic supplementary material.

ESM 1 (DOCX 17.5 KB)
