# Supplementary material for: Yellow meconium
Source: Forensic Sci Med Pathol. 2024 Dec 21;21(3):1561–5. doi: 10.1007/s12024-024-00932-2 (PMC12491340; doi:10.1007/s12024-024-00932-2)
Supplement: Supplementary file 1 — (DOCX 17.5 KB) [file 12024_2024_932_MOESM1_ESM.docx]

1. Adelson L (1974) The pathology of homicide. A vademecum for pa­thologist, prosecutor and defense counsel. CC Thomas, Spring­field Illinois
2. American College of Legal Medicine (2001) Legal medicine. Mosby, St. Louis/London/Philadelphia/Sydney
3. Berry CL (1989) Pediatric Pahtology. 2^nd^ Edition. Springer Verlag, London, Berlin, Heidelberg, New York,
4. Busuttil A, Keeling JW (2009) Paediatric forensic medicine and pa­thology. Hodder Arnold, London
5. Byard RW (2010) Sudden death in the young. Cambridge University Press, Cambridge
6. Camps FE (1976) Gradwohl’s legal medicine, 3. Aufl. John Wright & Sons Ltd., Bristol
7. Catanase CA (2010) Colour atlas of forensic medicine and patho­logy. CRC Press, Boca Raton
8. Collins KA, Byard RW (2014) Forensic pathology of infancy and childhood, Bd 2. Springer Science and Business Media, New York, Volumes 1-2
9. DiMaio DJ, DiMaio VJM (2001) Forensic pathology, 2. Aufl. Else­vier, New York
10. DiMaio VJM, Dana SE (1998) Vademecum Forensic Pathology. Landes, Austin
11. DiMaio VJM, Molina DK (2022) DiMaio’s Forensic Pathology. 3. Edition. CRC Press , Taylor & Francis Group, Boca Raton, London, New York
12. Dix J (2000) Colour atlas of forensic pathology. CRC Press, Boca Raton/London/New York
13. Dolinak D, Matshes E, Lew E (2005) Forensic pathology – principles and practice. Elsevier, Amsterdam/Boston/Heidelberg
14. Finkbeiner WE, Ursell PC, Davis RL (2004) Autopsy pathology – a manual and atlas. Churchill Livingstone, Philadelphia
15. Froede C (Hrsg) (2003) Handbook of forensic pathology, 2. Aufl. College of American Pathologists, Northfield
16. Garg S (2017) Pediatric pathology a course review. CRC Press Taylor & Francis Group, Boca Raton
17. Geberth VJ (2015) Practical homicide investigation. CRC-Press Tay­lor & Francis Group, Boca Raton
18. Gonzales TA, Vance M, Helpern M, Umberger C (1954) Legal medi­cine pathology and toxicology, 2. Aufl. Appleton-Century-Craft, New York
19. Gordon J, Shapiro HA, Berson SD (1988) Forensic medicine – a guide to principles, 3. Aufl. Churchill Livingstone, Edinburgh/ London/Melbourne
20. Houck MM (2017) Forensic pathology. Academic, Elsevier, London
21. Houck MM (2023) Encyclopedia of Forensic Sciences, 3. Edition, Elsevier Amsterdam, Boston, Heidelberg, London New York, Volume 1-4
22. Jamieson A, Moenssens A (Hrsg) (2009) Wiley encyclopaedia of fo­rensic sciences. Wiley, Chichester
23. Ludwig J (2004) Handbook of autopsy practice, 3. Aufl. Humana Press, Totowa
24. Madea B (2014) Handbook of forensic medicine. Wiley, Chichester
25. Madea B (2022) Handbook of Forensic Medicine. 2^nd^ Edition. Wiley Chichester, Volume 1-3
26. Mant AK (1984) Taylor’s principles and practice of medical juris­prudence, 13. Aufl. Churchill Livingstone, Edinburgh/London/ Melbourne/New York
27. Mason JK (1989) Paediatric forensic medicine and pathology. Chap­mann and Hall Medical, London
28. Mason JK (1993) Forensic medicine. An illustrated reference. Chap­man and Hall Medical, London/Glasgow/New York
29. Mason JK (1995) Forensic medicine for lawyers, 3. Aufl. Butter­worths, London
30. Payne-James J, Busuttil A, Smock W (2023) Forensic Medicine: Clinical and Pathological Aspects. GMM, San Francisco London
31. Payne-James J, Byard RW (2016) Encyclopedia of forensic and legal medicine, 4. Bd, 2. Aufl. Elsevier, Amsterdam/Boston
32. Payne-James J, Jones R (2020) Simpson’s forensic medicine, 14. Aufl. CRC Press, Taylor & Francis Group, Boca Raton
33. Payne-James J, Byard R, Corey RS, Henderson C (Hrsg) (2005) En­cyclopedia of forensic and legal medicine. Elsevier, Amsterdam
34. Payne-James J, Byard R (2024) Forensic and Legal Medicine. Clinical and Pathological Aspects. CRC Press, Taylor&Francis Group, Boca Raton, London, New York
35. Plueckhahn VD, Cordner S (1991) Ethics, legal medicine and foren­sic pathology, 2. Aufl. University Press, Melbourne
36. Polson CJ, Gee DJ, Knight B (1985) The essentials of forensic medi­cine, 4. Aufl. Pergamon Press, Oxford/New York/Toronto/Syd­ney/Paris/Frankfurt
37. Pomara C, Karch St B, Finechi V (2010) Forensic autopsy. Hand­book and atlas. CRC Press, Boca Raton
38. Prahlow J (2010) Forensic pathology for police, death investigators, attorneys and forensic scientists. Humana Press Springer, New York
39. Prahlow J, Byard R (2012) Atlas of forensic pathology. Humana Press, Dordrecht/Heidelberg/London
40. Rentoul E, Smith H (1973) Glaister’s medical jurisprudence and to­xicology, 13. Aufl. Churchill Livingstone, Edinburgh/London
41. Saukko P, Knight B (2004) Knight’s forensic pathology, 3. Aufl. Ed­ward Arnold, London
42. Saukko P, Knight B (2016) Knight’s forensic pathology, 4^th^ Edition. CRC Press, Taylor & Francis Group, Boca Raton, London, New York
43. Siegel JA, Saukko PK (2013) Encyclopedia of forensic sciences, 2. Aufl. Elsevier, Amsterdam
44. Siegel JA, Saukko PK, Knupfer GC (2000) Encyclopedia of forensic sciences, 3. Bd. Academic, San Diego/San Francisco/New York/ Boston/London/Sydney/Tokyo
45. Simpson K (1979) Forensic medicine, 8. Aufl. E Arnold Ltd, London
46. Spitz WU (1993) Spitz and Fisher’s Medicolegal Investigation of Death: guideline for the application of pathology to crime in­vestigation, 3. Aufl. Charles C Thomas Publ, Springfield
47. Spitz WU, Diaz FJ (2020) Medicolegal Investigation of Death. 5^th^ Edition, Charles C Thomas Publisher LTD, Springfield Illinois USA
48. Stocker J Th, Dehner L P (1992) Pediatric Pathology. J B Lippincott Company, Philadelphia, 2 Volumes
49. Tedeschi C, Eckert WG, Tedeschi LG (1977) Forensic medicine, 3. Bd. WB Saunders Company, Philadelphia/London/Toronto
50. Vanezis P (2020) Essential Forensic Medicine. Wiley Chichester
51. Whitwell H, Thorne K, Kolar A, Harvey P (2015) Mason’s forensic medicine for lawyers, 6. Aufl. Bloomsberry Professional, London
52. Wyatt J, Squires T, Norfolk G, Payne-James J (2011) Oxford hand­book of forensic medicine. Oxford University Press, Oxford
